# The etiology of diffuse cystic lung diseases: an analysis of 1010 consecutive cases in a LAM clinic

**DOI:** 10.1186/s13023-021-01905-2

**Published:** 2021-06-12

**Authors:** Han Cui, Chongsheng Cheng, Wenshuai Xu, Xinlun Tian, Yanli Yang, Yani Wang, Jiannan Huang, Yudi He, Jun Wang, Ruie Feng, Weihong Zhang, Kai-Feng Xu

**Affiliations:** 1 Department of Pulmonary and Critical Care Medicine, State Key Laboratory of Complex Severe and Rare Diseases, Peking Union Medical College Hospital, Chinese Academy of Medical Sciences, Beijing, China; 2 Department of Pathophysiology, State Key Laboratory of Medical Molecular Biology, Institute of Basic Medical Sciences, Chinese Academy of Medical Sciences, Peking Union Medical College, Beijing, China; 3 Department of Pathology, Peking Union Medical College Hospital, Chinese Academy of Medical Sciences, Beijing, China; 4 Department of Radiology. Peking, Union Medical College Hospital, Chinese Academy of Medical Sciences, Beijing, China

**Keywords:** Diffuse cystic lung diseases, Etiology, Differential diagnosis, Lymphangioleiomyomatosis, Pulmonary Langerhans cell histiocytosis, Birt–Hogg–Dubé syndrome, Sjogren’s syndrome

## Abstract

**Background:**

The differential diagnosis of diffuse cystic lung disease (DCLD) is a clinical challenge. We wish to analyze the distribution of the etiology of DCLD based on data from a single lymphangioleiomyomatosis (LAM) clinic.

**Methods:**

All DCLD patients at the LAM Clinic of Peking Union Medical College Hospital between January 2006 and December 2019 were analyzed. Information on the demographic, clinical, radiological, and pathological features was collected.

**Results:**

A total of 1010 patients with DCLD on CT scan were evaluated. A sum of 711(70.4%) patients were diagnosed with definite or probable LAM. Other diagnoses included Birt–Hogg–Dubé syndrome (46), Sjogren's syndrome (38), pulmonary Langerhans cell histiocytosis (14), lung tumors (3), Castleman disease (2), antineutrophil cytoplasmic antibody-associated vasculitis (2), systemic lupus erythematosus (1), Marfan syndrome (1), amyloidosis (1), congenital cystic adenomatoid malformation of the lung (1), and pleuroparenchymal fibroelastosis (1). In the 38 patients diagnosed with Sjogren's syndrome, 2 were diagnosed with light-chain deposition disease, 2 were diagnosed with amyloidosis and 1 was diagnosed with lymphocytic interstitial pneumonia. One hundred and eighty-nine patients (18.7%) were undiagnosed. Lung biopsy results were available in 27 patients in the undiagnosed DCLD group but did not provide a diagnosis.

**Conclusion:**

Approximately 70% of DCLD patients in our LAM clinic had LAM. The common differential diagnoses included Birt–Hogg–Dubé syndrome, Sjogren’s syndrome, and pulmonary Langerhans cell histiocytosis. Detailed clinical information and laboratory, genetic, and pathological investigations provide correct diagnoses in most patients with DCLD.

## Introduction

Diffuse cystic lung disease (DCLD) refers the presence of multiple air-filled spaces with a wall thickness less than 2 mm and clear boundaries with the lung tissues [1]. An increasing number of diseases labeled DCLD make the differential diagnosis of this condition difficult [2]. The clinical features and radiological findings are quite similar in many patients. An exploration of the appropriate diagnosis is a great clinical challenge. The aim of this study was to analyze the clinical data of DCLD patients registered in the Lymphangioleiomyomatosis (LAM) clinic of Peking Union Medical College Hospital (PUMCH) in recent years. We summarized the distribution of their etiologies and proposed a stepwise diagnostic procedure to improve the diagnosis of DCLD.

## Materials and methods

### Subjects

All DCLD patients presented in the LAM Clinic of PUMCH between January 2006 and December 2019 were retrospectively analyzed. Their lung CT scans met the criteria for diffuse pulmonary cystic lesions: at least 3 air-filled spaces with a cyst wall less than 2 mm, clearly demarcated from normal lung tissue. Patients with incomplete data were excluded. The study was approved by the Ethical Committee of PUMCH (S-K1583).

### Diagnosis

The diagnostic criteria of LAM were initially based on the guidelines of the European Respiratory Society (ERS) in 2010 [3] and updated according to the 2017 guidelines by the American Thoracic Society and Japanese Respiratory Society (ATS/JRS) [4]. All definite LAM cases were reviewed and diagnosed based on the 2017 guidelines. Probable LAM was diagnosed based on the criteria of the 2010 guidelines, without other supporting evidence. The diagnoses of Sjogren's syndrome (SS) [5], Birt–Hogg–Dubé syndrome (BHD) [6], pulmonary Langerhans cell histiocytosis (PLCH) [7], systemic lupus erythematosus [8], Castleman’s disease [9], antineutrophil cytoplasmic antibody-associated vasculitis [10], and Marfan syndrome [11] were based on published criteria. Amyloidosis, light-chain deposition disease (LCDD), congenital cystic adenomatoid malformation of the lung, pleuroparenchymal fibroelastosis, and lung tumor were diagnosed based on pathology.

## Results

### Patient population

A total of 1076 patients with diffuse cystic changes on HRCT of the chest at the PUMCH LAM Clinic between January 2006 and December 2019 were included. Sixty-six subjects were excluded because of insufficient clinical information. A total of 1010 patients were finally analyzed in this article.

### Etiology

In our LAM clinic, most subjects (711/1010, 70.4%) were diagnosed with LAM. Among them, the number of individuals with definite and probable LAM was 646 and 65, respectively. Most of the cases were sporadic LAM, and 58 were tuberous sclerosis (TSC)-associated LAM. Pathological results were obtained in 38.1% of LAM patients. The remaining 65 cases were probable LAM, 47 of them have serum vascular endothelial growth factor-D (VEGF-D) tested and within normal ranges.

The number of SS patients was 38. Two patients were diagnosed with light-chain deposition disease (LCDD) and 2 patients were diagnosed with amyloidosis by video-assisted thoracic surgery (VATS) or transbronchial lung biopsy (TBLB). One patient was diagnosed with lymphocytic interstitial pneumonia (LIP) by VATS. There were 46 patients diagnosed with BHD, all of whom had folliculin (*FLCN*) gene mutations. Fourteen patients were diagnosed with PLCH. These PLCH patients underwent VATS lung biopsy or TBLB, and the sample showed Langerhans cells. Three patients had lung tumors, and their pathology results were lung adenocarcinoma (1 patient), lymphangioma (1 patient), and unclassified spindle cell tumors (1 patient). Two patients had Castleman disease based on lung biopsy, clinical manifestations, and laboratory tests. Two patients were diagnosed with antineutrophil cytoplasmic antibody-associated vasculitis based on manifestations, lung CT findings, and positive for anti-neutrophil cytoplasmic antibody (ANCA). One patient had Marfan syndrome based on typical clinical features and a family history of Marfan syndrome and a mutation of *FBN1*. One patient was diagnosed with systemic lupus erythematosus based on multisystemic involvement and positivity of anti-double-stranded DNA. One patient with amyloidosis, one patient with congenital cystic adenomatoid malformation of the lung, and one patient with pleuroparenchymal fibroelastosis were diagnosed by lung biopsy. For the other 189 patients, a definite diagnosis could not be made after several examinations. The distribution of etiologies is shown in Table 1. The top four causes of DCLD were LAM, BHD, SS, and PLCH, accounting for 80.1% of the causes of DCLD. There were 18.7% of patients undiagnosed.

**Table 1:** Etiology of diffuse cystic lung diseases

Causes	Numbers (%)
Lymphangioleiomyomatosis	711 (70.4%)
Birt–Hogg–Dubé syndrome	46 (4.6%)
Sjogren syndrome	38 (3.8%)
Light-chain deposition disease	2
Amyloidosis	2
Lymphocytic interstitial pneumonitis	1
Pulmonary Langerhans cell histiocytosis	14 (1.4%)
Tumor	3 (0.3%)
Castleman’s disease	2 (0.2%)
Antineutrophil cytoplasmic antibody-associated vasculitis	2 (0.2%)
Systemic lupus erythematosus	1 (0.1%)
Marfan syndrome	1 (0.1%)
Amyloidosis*	1 (0.1%)
Congenital cystic adenomatoid malformation of the lung	1 (0.1%)
Pleuroparenchymal fibroelastosis	1 (0.1%)
Undiagnosed diffuse cystic lung diseases	189 (18.7%)
Total	1010 (100%)

^*^This case of amyloidosis did not have Sjogren’s syndrome

### Basic information of the DCLD patients

Clinical features including age, sex, smoking history, and family history among the different etiologies are shown in Table 2. Most patients were female at our LAM Clinic except for patients with PLCH. A total of 92.9% (13/14) of PLCH patients had a smoking history. Family history was suggested in 1.1% (8/711) of LAM patients (all of them were TSC-associated) and in 80.4% (37/46) of BHD patients.

**Table 2:** Demographic characteristics among different causes of DCLD

	Numbers	Age (years)	Female (%)	Smoking history	Family history
LAM	711	38.2 ± 10.8	711(100.0%)	1.1%	1.1%
BHD	46	47.3 ± 10.4	42 (91.3%)	6.5%	80.4%
SS	38	49.7 ± 10.4	38 (100.0%)	2.6%	5.3%
PLCH	14	32.8 ± 12.4	6 (42.9%)	92.9%	0
Tumor	3	41–50	3 (100.0%)	0	0
CD	2	34–46	1 (50%)	50%	0
AVA	2	37–51	2 (100%)	0	0
SLE	1	45	1 (100%)	0	0
MFS	1	57	0	0	100.0%
AMY	1	32	1 (100.0%)	0	0
CCAM	1	28	1 (100.0%)	0	0
PPFE	1	37	1 (100.0%)	0	0

*AMY* amyloidosis, *AVA* antineutrophil cytoplasmic antibody-associated vasculitis, *BHD* Birt–Hogg–Dubé syndrome, *CCAM* congenital cystic adenomatoid malformation of the lung, *CD* Castleman disease, *DCLD* diffuse cystic lung disease, *LAM* lymphangioleiomyomatosis, *MFS* Marfan syndrome, *PPFE* pleuroparenchymal fibroelastosis, *PLCH* pulmonary Langerhans cell histiocytosis, *SS* Sjogren's syndrome

### Laboratory tests and other evaluations

The characteristic clinical manifestations and laboratory tests are shown in Table 3. Among the patients diagnosed with LAM, 35.0% had pneumothorax, 30.2% had renal angiomyolipoma, 17.4% had chylothorax, and 3.9% had chylous ascites. Among patients with SS, 78.9% experienced dry mouth, and 65.8% experienced dry eye. The positive rate of anti-SSA or anti-SSB antibody was 97.4%. A total of 52.2% of BHD patients showed pneumothorax, and 17.4% showed skin involvement. The *FLCN* gene mutation were found in all BHD patients. Three patients with PLCH had diabetes insipidus, and magnetic resonance imaging of the head indicated pituitary funnel lesions. The patient with systemic lupus erythematosus showed symptoms with multisystem involvement and anti-dsDNA antibody positivity. The patient with Marfan syndrome was from a Marfan syndrome family, with a thin body habitus, long limbs, and an *FBN1* gene mutation. One patient's laryngoscope revealed a larynx mass, and the pathology was amyloidosis. The patient with ANCA-associated vasculitis was ANCA positive. No clinical manifestation was recorded for the patients with congenital cystic adenomatoid malformation of the lung and pleuroparenchymal fibroelastosis.

**Table 3:** Characteristic clinical manifestations and laboratory tests of DCLD

	Clinical manifestations	Evaluations
LAM	Pneumothorax 35.0%, renal angiomyolipoma 30.2%, chylothorax 17.4%, chylous ascites 3.9%, TSC 8.2%	VEGF-D ≥ 800 pg/ml 89.0%
SS	Dry mouth 78.9%, dry eyes 65.8%	Anti-SSA or anti-SSB antibody positive 97.4%
BHD	Pneumothorax 52.2%, fibrofolliculomas or trichodiscomas 17.4%	*FLCN* gene mutation 100%
PLCH	Pneumothorax 28.6%, Diabetes insipidus 7.1%	MRI showed pituitary funnel lesions 21.4%

*BHD* Birt–Hogg–Dubé syndrome, *LAM* lymphangioleiomyomatosis, *MRI* magnetic resonance imaging, *PLCH* pulmonary Langerhans cell histiocytosis, *SS* Sjogren's syndrome, *VEGF-D* vascular endothelial growth factor-D

### Undiagnosed DCLD

One hundred eighty-nine patients (174 females, 15 males) were classified as having undiagnosed DCLD. Twelve of them had a smoking history. They underwent several examinations but still did not receive a diagnosis. Ten patients were consulted in the stomatology department without meaningful results. Ophthalmology tests were performed in 27 patients, and 5 patients were diagnosed with dry eye disease. Among the undiagnosed patients, serum protein electrophoresis (102 patients tested), immunofixation electrophoresis (123 tested), anti-SSA/SSB (135 tested), and ANCA (128 tested) were performed with negative results. Abdominal ultrasound was performed in 60 patients. Abdominal CT examination was performed in 45 patients, and kidney nodules were found in 1 patient. *FLCN* gene tests were performed in 29 patients, all of which were negative. One hundred forty-two VEGF-D examinations were performed, and all were lower than 800 pg/ml. Twenty-six lung pathological biopsies were taken including 16 TBLB and 10 VATS. None of pathological results were suggestive. Among these 26 patients who underwent lung biopsy, 2 patients underwent *FLCN* gene test with negative results.

## Discussion

In this group, the most common causes of DCLD were LAM, SS, BHD, and PLCH, accounting for 80.1% of all causes. 18.7% were undiagnosed.

Research on the etiological distribution of DCLD is very limited; however, it is believed that the major causes of DCLD include LAM, PLCH, LIP, and BHD [12]. SS has not been listed sometimes because multiple pathological bases were found, such as LIP. Other causes of pathological abnormality in cystic lung lesion in patients with SS includes nonspecific interstitial pneumonia, usual interstitial pneumonia, organizing pneumonia, pulmonary lymphoma, pulmonary amyloidosis, and so on [13, 14]. Among the patients in this study, based on data available, 2 were diagnosed with amyloidosis, 2 were diagnosed with LCDD, and 1 was diagnosed with LIP. It seems that LIP was not the most common type of pathological abnormality.

Sex, smoking history, and family history are important in suggesting a differential diagnosis. Almost all LAM patients were women of childbearing age, and family history was mainly related to BHD and TSC. BHD is an autosomal-dominant genetic disease. A total of 80.4% of patients had a family history of pneumothorax or bullae in this report. A total of 57.1% of PLCH patients were male, and 92.9% of them had a history of smoking and no family history of similar diseases. All the SS patients were female in this report.

In the differential diagnosis of DCLD, characteristic changes in cysts in the lung can suggest a diagnosis clue of LAM type, BHD type, PLCH type, or others [2]. Laboratory investigations have mainly focused on autoimmune diseases such as SS and LCDD or amyloidosis. Recently, VEGF-D has been used in the diagnosis of LAM [15, 16]. Gene tests, for single gene (FLCN, or TSC1/TSC2), DCLD gene panel, or whole exome sequencing were selected depending on the aims of the test applied.

In our LAM clinic, LAM was the most common cause of DCLD. LAM can be diagnosed based on history (chylothorax, renal AML, or TSC), VEGF-D, and pathology [4]

Three patients in this group were diagnosed with tumors by a pathological diagnosis of their pulmonary cysts. Tumors that cause cystic lesions in the lung are mainly metastatic sarcoma and tumors derived from mesenchymal cells, including lung adenocarcinoma, bronchioloalveolar carcinoma, lymphoma, malignant vascular endothelial cell tumor, and osteosarcoma [17]. In this study, the pathology results of the 3 tumor patients were lung adenocarcinoma, lymphangioma and spindle cell tumor.

In this group, 189 patients had been labeled DCLD with unknown causes, accounting for a large proportion. Although they were screened initially, many of them lacked some tests, mainly either genetic screens or lung biopsies.

**Fig. 1:**
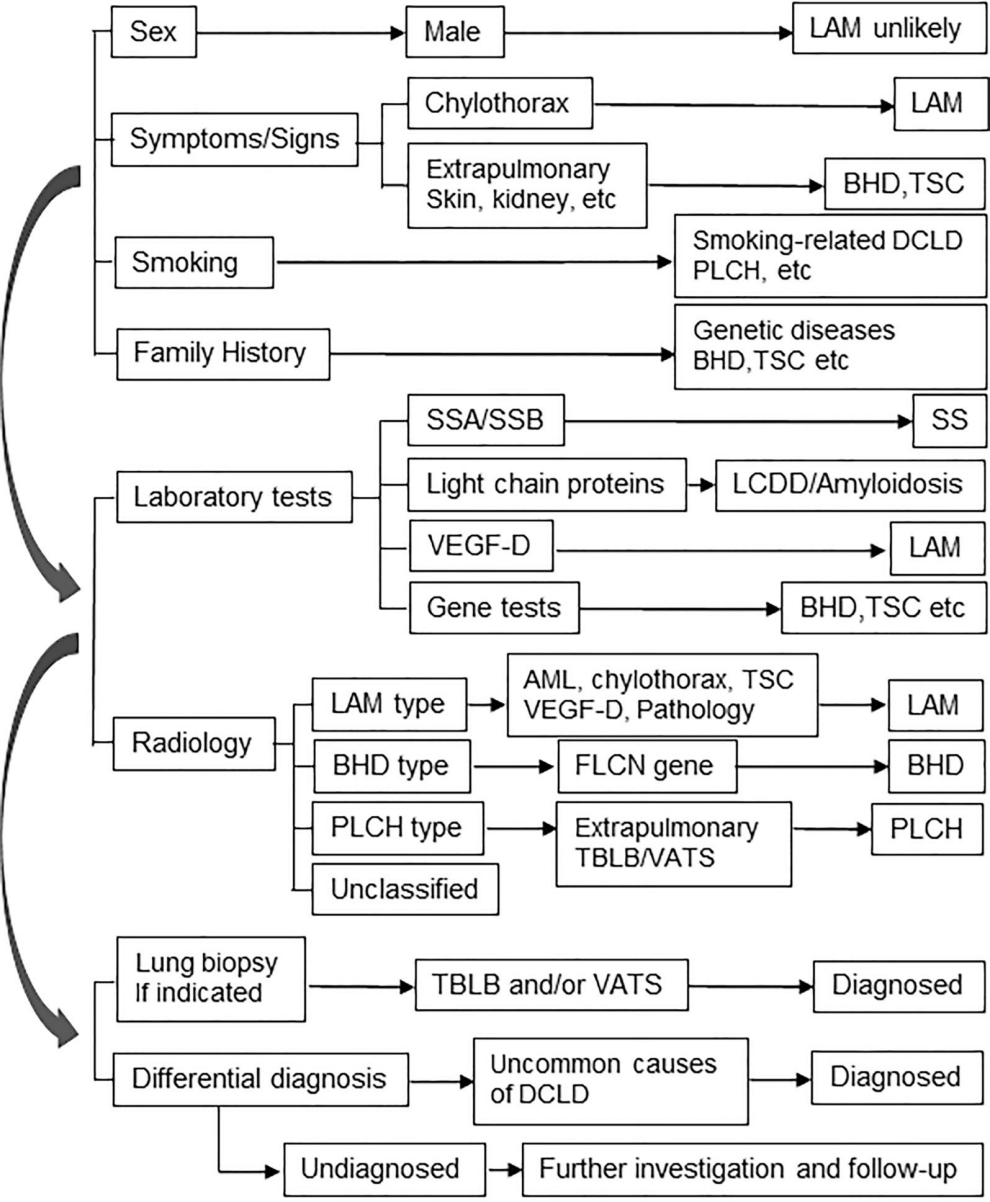
Stepwise diagnostic procedure for common causes of diffuse cystic lung diseases. *BHD* Birt–Hogg–Dubé syndrome, *FLCN* folliculin, *LAM* lymphangioleiomyomatosis, *LCDD* light chain deposition disease, *PLCH* pulmonary Langerhans cell histiocytosis, *SS* Sjogren's syndrome, *SSA/SSB* Sjogren's syndrome antibodies A and B, *TBLB* transbronchial biopsy, *TSC* tuberous sclerosis complex, *VATS* video-assisted thoracoscopy, *VEGF-D* vascular endothelial growth factor-D

**Fig. 2:**
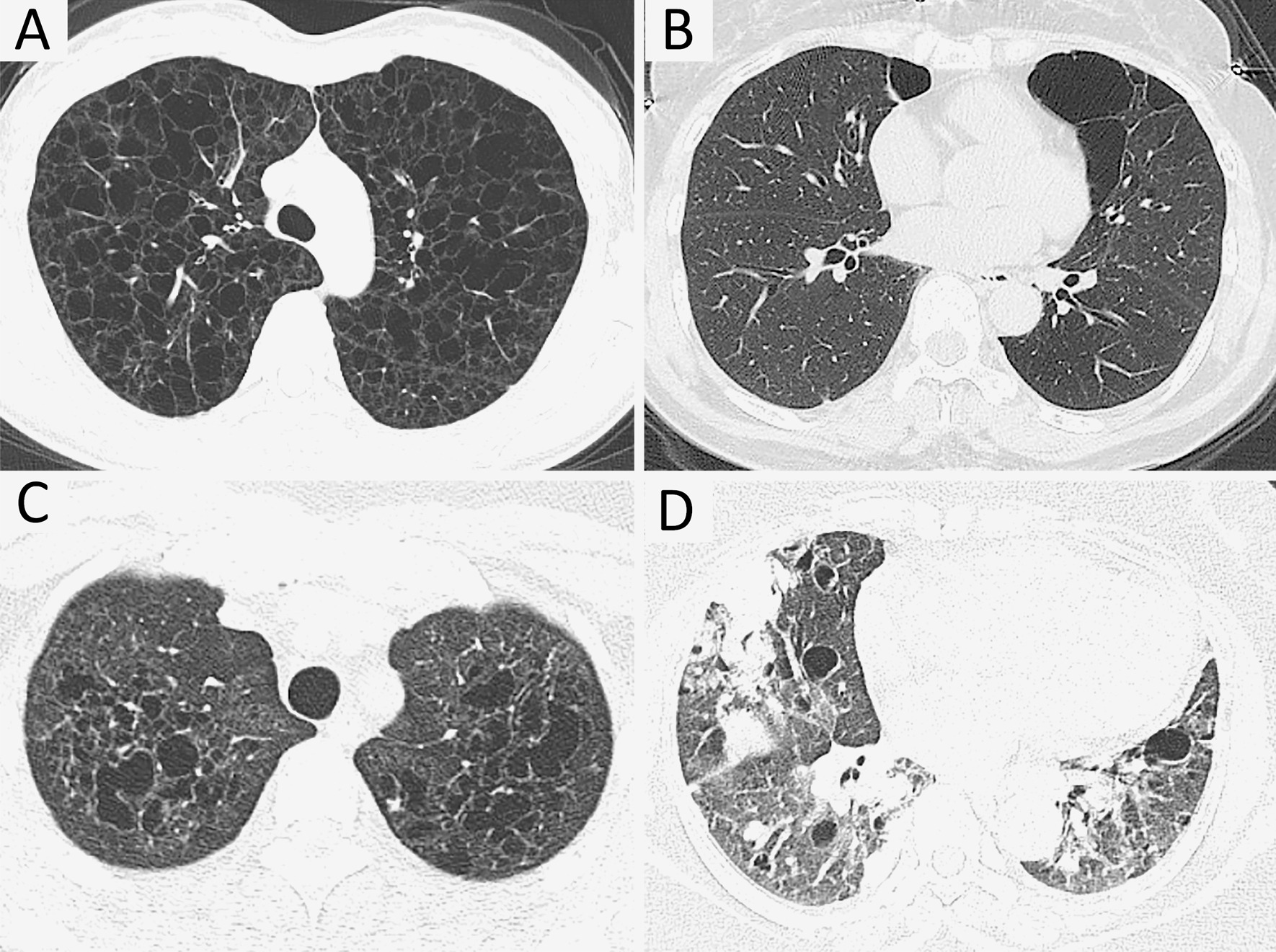
Typical images of DCLD on CT scans are suggestive of a diagnosis. **A** A typical lymphangioleiomyomatosis (LAM) image in a 38-year-old woman shows a evenly distributed diffuse thin-walled cysts crossover all lung fields. Pneumothorax and chylothorax are frequently seen (not shown) in patients with LAM. (2) A typical Birt–Hogg–Dubé syndrome image in a 63-year-old woman shows large and irregular cysts frequently distributed in the lower lung fields and subpleural or paramediastinal regions. (3) A typical pulmonary Langerhans cell histiocytosis (PLCH) image in a 32-year-old woman with a history of cigarette smoking shows upper and middle lung field dominant distribution with irregular cysts and multiple nodules. Costophrenic regions are likely less involved (not shown). (4) A 66-year-old women with a history of Sjogren syndrome was found diffuse cystic changes in the lung. Images on CT show randomly distributed cysts that are larger than cysts usually presented in LAM as well as patchy infiltrates and nodules. Light chain deposition disease was diagnosed after thoracoscopic lung biopsy

Based on the most common causes of DCLD, we proposed a stepwise diagnosis approach (Fig. 1). A detailed medical history, physical examination (sex, symptoms/signs, smoking history, and family history) and pulmonary HRCT should be taken and analyzed to look for clues to common DCLD. Typical images of cysts on HRCT are suggestive of a diagnosis (Fig. 2), and are clues of further investigations. Further laboratory tests, gene tests and lung biopsy are frequently required. For undiagnosed DCLD, further diagnosis and follow-up are essential.

There are some limitations of our study. The patients in the LAM clinic were mainly female and showed signs suggestive of LAM; therefore, there was a selection bias among the patients in this report. The data represented were from only one clinic and, therefore, may not be representative of other populations. additionally, in the patients with undiagnosed DCLD, the gene tests and lung biopsy were inadequate.

We concluded that approximately 70% of DCLD patients presenting to the LAM clinic were diagnosed with LAM. Common causes in the differential diagnosis included SS, BHD, and PLCH. Detailed clinical information and laboratory, genetic, and pathological investigations can provide correct diagnoses in most patients with DCLD.

## Data Availability

The dataset used in this research and analysis were available from the corresponding authors.

## Abbreviations

AML: angiomyolipoma
AMY: amyloidosis
ANCA: anti-neutrophil cytoplasmic antibody
AVA: antineutrophil cytoplasmic antibody-associated vasculitis
BHD: Birt–Hogg–Dubé syndrome
CCAM: congenital cystic adenomatoid malformation of the lung
CD: Castleman disease
DCLD: diffuse cystic lung disease
LAM: lymphangioleiomyomatosis
LCDD: light chain deposition disease
LIP: lymphocytic interstitial pneumonia
MFS: Marfan syndrome
MRI: magnetic resonance imaging
PPFE: pleuroparenchymal fibroelastosis
PLCH: pulmonary Langerhans cell histiocytosis
PUMCH: Peking Union Medical College Hospital
SS: Sjogren's syndrome
SSA/SSB: Sjogren's syndrome antibodies A and B
TBLB: transbronchial lung biopsy
TSC: tuberous sclerosis complex
VATS: video-assisted thoracic surgery
VEGF-D: vascular endothelial growth factor-D
